# A Case of Metastatic Synovial Sarcoma of the Lung With Recurrent Spontaneous Pneumothorax

**DOI:** 10.1002/cnr2.70104

**Published:** 2025-02-02

**Authors:** Xunlai Lu, Shengfu Liu, Luyao Ma, Junwei Zhu, Wei Cao

**Affiliations:** ^1^ Department of Cardiovascular Surgery Northeast Yunnan Central Hospital Zhaotong China

**Keywords:** lung, metastatic, spontaneous pneumothorax, synovial sarcoma

## Abstract

**Background:**

Synovial sarcoma, a highly aggressive soft tissue sarcoma, is mostly seen among young people and tends to occur in the extremities. It most commonly metastasizes to the lungs, and symptoms such as chest tightness, shortness of breath, and coughing are common after metastasis. However, multiple spontaneous pneumothorax is relatively rare. This article aims to further enhance the understanding of synovial sarcoma by introducing a case of pulmonary metastasis after surgery for synovial sarcoma originating from the sole of the foot and reviewing relevant literature.

**Case:**

The Cardiothoracic Surgery Department of the Northeast Yunnan Central Hospital Region admitted a 40‐year‐old male patient. He had previously undergone surgery for synovial sarcoma on the left foot dorsum. Seven years after the surgery, the patient experienced two spontaneous pneumothorax events. A chest CT scan revealed solid nodules in the upper lobe of the left lung. Our department performed surgical removal, and pathology confirmed metastatic pulmonary synovial sarcoma. After the surgery, the patient's symptoms improved significantly, and there were no further pneumothorax attacks. The patient was then transferred to the oncology department for further treatment to improve prognosis.

**Conclusion:**

Pulmonary metastasis of synovial sarcoma is not uncommon, and a considerable number of patients are found to have no surgical opportunity when diagnosed. In this case, the patient presented with a single solid nodule, and we adopted surgery as the first‐line treatment, combined with a comprehensive treatment plan of postoperative chemotherapy to further improve the patient's prognosis.

## 1 Introduction

The imaging manifestations of metastatic pulmonary synovial sarcoma (SS) patients are nonspecific, and diagnosis relies on clinical history and pathology. We treated a patient who had previously undergone resection of a biphasic SS on the left foot. Seven years later, the patient experienced multiple episodes of pneumothorax. A chest CT scan revealed a single solid nodule of 18 mm × 12 mm in the upper lobe of the left lung. The patient had no history of emphysema, tuberculosis, or lung cancer. Therefore, we considered the possibility of metastatic pulmonary SS and could not rule it out. The pathological results after thoracoscopic surgery confirmed our suspicion. In our view: For spontaneous pneumothorax caused by pulmonary metastasis of SS, thoracoscopic surgery can improve symptoms. It can reduce the recurrence rate of pneumothorax. Thoracoscopic surgery can also enhance the patient's quality of life and improve prognosis. The pathological findings after thoracoscopic surgical removal confirmed our diagnosis, further supporting the effectiveness of this approach in managing such cases [1].

## 2 Case Presentation

A 40‐year‐old male patient with a 15‐year smoking history of 15 cigarettes per day. Initially, in 2011, he experienced intermittent pain in his left foot without an apparent cause. The pain intensified when stepping on foreign objects or being squeezed. He was treated with local closed therapy in an external hospital in August 2011, which temporarily relieved his symptoms. However, the pain relapsed half a year later, accompanied by the appearance of a 2 × 1.5 cm oval‐shaped mass on the left foot. Despite multiple conservative treatments at external hospitals, there was no significant improvement. In September 2014, the patient underwent left foot mass resection and pathological examination, which confirmed the diagnosis of biphasic SS. He was then treated with three cycles of doxorubicin combined with ifosfamide chemotherapy in July 2023, the patient developed left chest pain without an apparent cause. He was diagnosed with spontaneous pneumothorax and treated with closed thoracic drainage. Unfortunately, the pneumothorax relapsed in September 2023, and a chest CT scan revealed a solid nodule in the upper lobe of the left lung.

**FIGURE 1:**
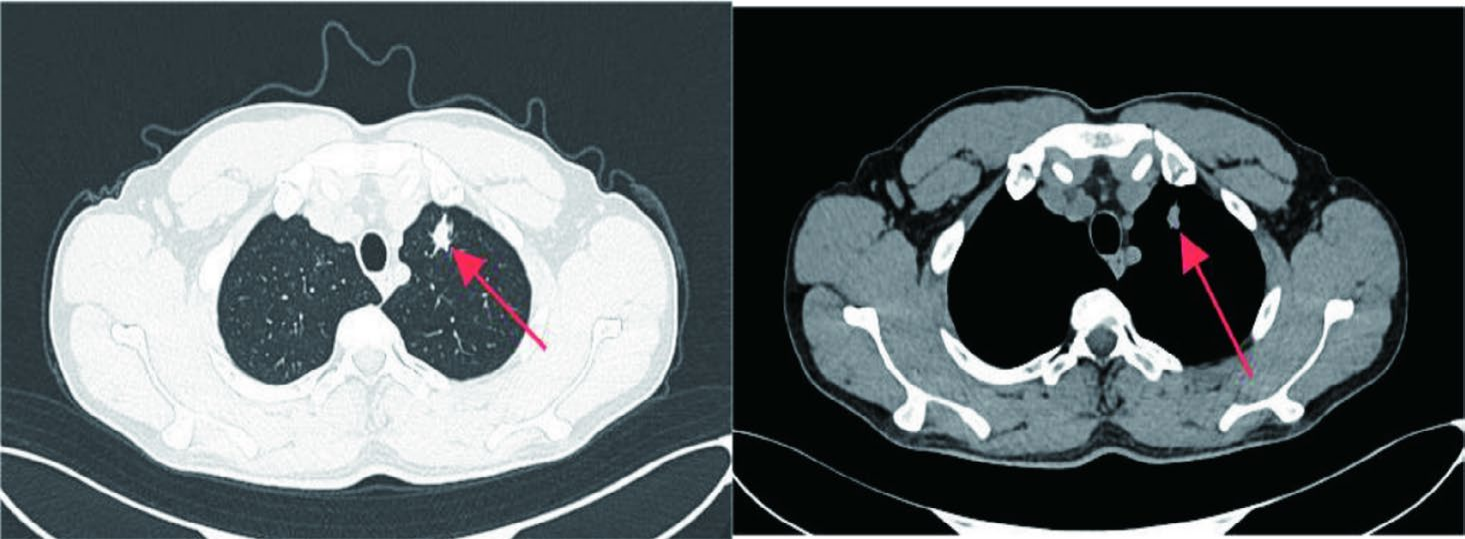
The patient's chest CT scan revealed a solid nodule (red arrow) at the apicoposterior segment of the upper left lobe of the lung, located close to the pleura, measuring approximately 1.8 × 1.2 cm. There are visible spiculated signs around the nodule, and no calcification was observed on the mediastinal window.

**FIGURE 2:**
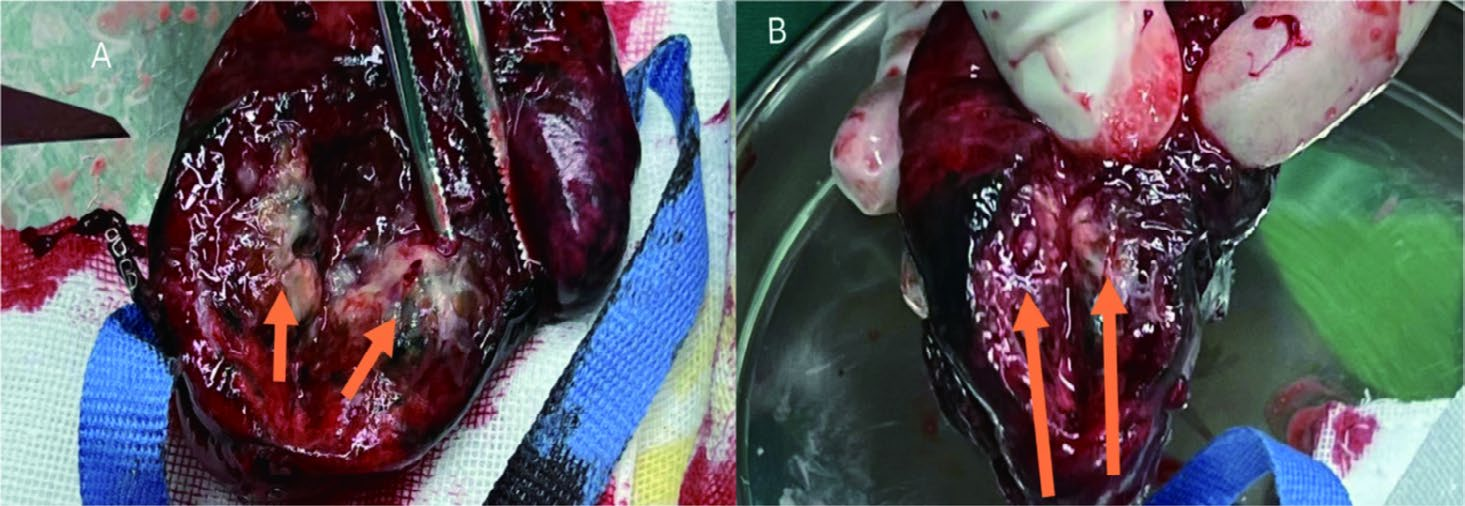
This is the specimen resected during the surgery. It can be seen that the whole tumor was resected intact, with a size of about 1.8 cm × 1.5 cm, grayish white in color, and obvious tissue necrosis (orange arrow).

**FIGURE 3:**
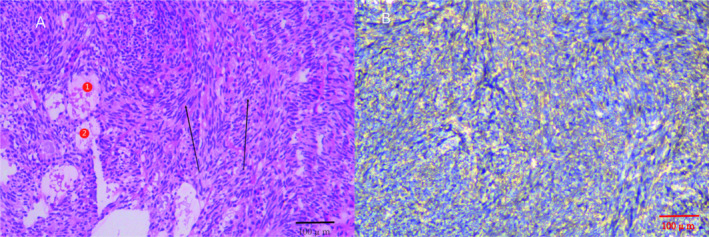
On this pathological slide, spindle‐shaped cells with relatively consistent morphology were arranged in bundles (black arrow). Epithelial‐like cells were columnar and glandularin structure, and eosinophilic secretions were visible (red numbers). The diagnosis was biphasic synovial sarcoma (HE original magnification ×100).

At our hospital, chest CT showed an 18 mm × 12 mm solid nodule with spiculated edges in the upper left lung lobe (Figure 1). After a careful evaluation of the patient's history, imaging, and clinical presentation, metastatic pulmonary SS was suspected. Although the patient refused puncture biopsy and PET‐CT, he strongly requested surgical treatment. In October 2023, thoracoscopic wedge resection of the upper lobe of the left lung was performed, and the tumor was completely removed with negative margins (Figure 2). Intraoperative frozen section analysis hinted at biphasic SS, which was consistent with the preoperative evaluation. Postoperative pathology and immunohistochemistry further confirmed the diagnosis, with results including Desmin (−), Myogenin (−), Caldesmon (−), SMA (−), SDHB (+), Ki‐67 (40%), CD99 (+), Bcl‐2 (+), AE1/AE3 (+), EMA (+), Calponin (+), CD34 (vascular +), S100 (−), and CD117 (−) (Figure 3).

**FIGURE 4:**
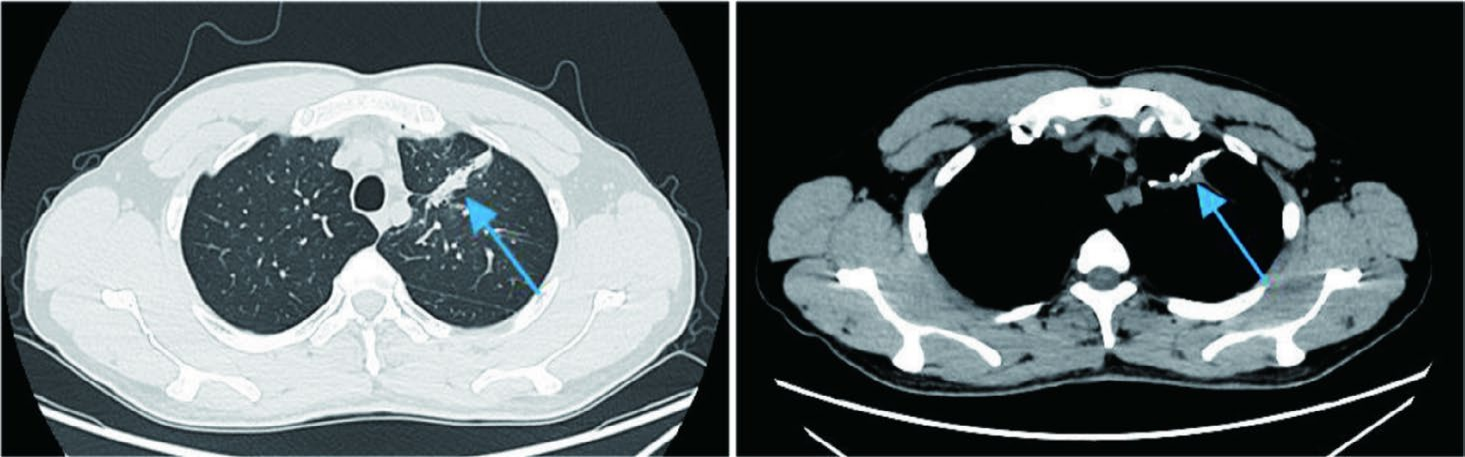
The nodule is close to the periphery, so wedge resection is chosen during the operation. After the operation, the CT scan shows the shadow of the stapler chain. The tumor is resected with R0 resection while maximizing the preservation of lung function. The lung expands completely after the operation, and the postoperative recovery is good.

The postoperative recovery was good, and the chest CT showed that lung function had been maximally preserved (Figure 4). The patient subsequently received four cycles of doxorubicin combined with ifosfamide chemotherapy. A half‐year follow‐up showed no tumor recurrence, no pneumothorax attacks, and a significant improvement in quality of life.

## 3 Discussion

SS is a rare, highly aggressive mesenchymal malignancy with an incidence rate ranking fourth among soft tissue sarcomas [2]. It mostly occurs in young people and is common in the extremities, often metastasizing to the lungs and surrounding pleura. Clinically, it often manifests as cough, expectoration, dyspnea, and other discomforts [3]. The incidence of spontaneous pneumothorax is only 1.9% [4]. Currently, there are three hypothesized mechanisms in the literature for how malignancies can cause pneumothorax. First, pneumothorax may be caused by the spontaneous rupture of necrotic tumors, leading to the formation of bronchopleural fistula. Second, excessive expansion of peripheral lung tumors may result in the formation and rupture of subpleural bullae. Finally, tumor spread to the pleura itself may cause pleural rupture and the formation of pneumothorax [5]. The appearance of spontaneous pneumothorax may indicate the aggressiveness or late stage of the disease, with a poor prognosis [6].

Metastatic pulmonary SS is mainly differentiated from primary lung cancer, hamartoma, tuberculoma, and other soft tissue sarcomas of the lung and pleura, such as fibrosarcoma, rhabdomyosarcoma, and leiomyosarcoma. Imaging examination provides important reference value for diagnosis. CT images of metastatic pulmonary SS mostly show round or quasi‐circular nodules or mass shadows of varying sizes, with clear boundaries and uniform density [6]. If the lung mass is obvious and accompanied by hilar and mediastinal lymph node enlargement, it is more likely to be diagnosed as primary lung cancer. SS is characterized by the chromosomal translocation of t(X;18)(p11.2;q11.2), forming the SYT‐SSX fusion gene, which is the gold standard for diagnosis [7]. It can be definitively diagnosed through cytogenetics, FISH gene detection, or RT‐PCR detection. Pyden and Lin [8]. compared the diagnostic value of immunohistochemistry and molecular biology methods and believed that cases that can be diagnosed using traditional diagnostic methods do not require SYT‐SSX testing. In this case, the diagnosis has been confirmed by immunohistochemistry, so these tests were not performed.

Surgery is considered the first‐line treatment for metastatic SS [9], and its effectiveness is influenced by factors such as tumor size, location, staging, and resection scope [10, 11]. SS is sensitive to chemotherapy [3], and neoadjuvant therapy has been proven to improve survival rates. The use of chemotherapy has also been reported as a risk factor for the development of spontaneous pneumothorax [6]. Chemotherapy drugs such as doxorubicin and ifosfamide are commonly used to help control distant metastases, while pazopanib can be used if chemotherapy drugs fail [2].

The patient in this case had surgical removal of a primary SS on the left foot 9 years ago but did not receive standardized treatment and regular follow‐up after the surgery. Nine years later, the tumor recurred, and the patient experienced spontaneous pneumothorax multiple times, suggesting a possible poor prognosis. It also reminds us to be vigilant about spontaneous pneumothorax, which may be the first manifestation of metastatic disease that is not detected by imaging. Although some patients can be managed through bed rest, closed thoracic drainage, and other methods [12], untreated pneumothorax often recurs. Considering that the tumor in this patient was localized and small, providing an opportunity for complete surgical removal, we performed a left upper lobe wedge resection assisted by a single‐port thoracoscopy at the fifth intercostal space on the left side, combined with closed thoracic drainage. The tumor was removed with R0 resection, while maximizing the preservation of lung function. The patient recovered smoothly after the surgery, with the chest drainage tube removed on the second day and discharged on the fifth day. After discharge, the patient received four cycles of combination chemotherapy with doxorubicin and ifosfamide in the oncology department. Since the surgery, there has been no recurrence of pneumothorax or tumor. One prospective study showed almost 50% of deaths occurred within 5–10 years of diagnosis, emphasizing the importance of long‐term follow‐up [2]. We will also continue to track the patient's subsequent disease progression.

## 4 Conclusion

In summary, the diagnosis of metastatic pulmonary SS requires a combination of medical history, imaging, and pathology. Currently, there is no standard guideline for treatment [13], and treatment plans should be individualized as much as possible. Comprehensive treatment through a combination of surgery, chemotherapy, radiotherapy, and immunotherapy can achieve the best prognosis for patients. It is foreseeable that in the future, comprehensive treatment will provide greater help to patients.

## Author Contributions

**Xunlai Lu:** conceptualization, data curation, formal analysis, investigation, methodology, project administration, validation, writing – original draft, writing – review and editing. **Shengfu Liu** and **Luyao Ma:** data curation, investigation, methodology, writing – review and editing. **Junwei Zhu:** methodology, writing – review and editing. **Wei Cao:** conceptualization, data curation, formal analysis, investigation, methodology, project administration, supervision, validation, writing – original draft, writing – review and editing.

## Ethics Statement

The studies involving human participants were reviewed and approved by Institutional Review Board of Northeast Yunnan Central Hospital.

## Consent

Written informed consent was obtained from all the participants prior to the enrollment (or for the publication) of this case report.

## Conflicts of Interest

The authors declare no conflicts of interest.

## Data Availability

The original contributions presented in the study are included in the article, Further inquiries can be directed to the corresponding author.
